# Dynamically patterning x-ray beam by a femtosecond optical laser

**DOI:** 10.1126/sciadv.adp5326

**Published:** 2024-11-20

**Authors:** Kenji Tamasaku, Takahiro Sato, Taito Osaka, Hitoshi Osawa, Diling Zhu, Tetsuya Ishikawa

**Affiliations:** ^1^ RIKEN SPring-8 Center, Sayo-cho, 679-5148, Japan.; ^2^ Japan Synchrotron Radiation Research Institute, Sayo-cho, 679-5198, Japan.; ^3^ Linac Coherent Light Source, SLAC National Accelerator Laboratory, Menlo Park, CA 94025, USA.

## Abstract

Modern science and technology have greatly benefitted from our ability to precisely manipulate light waves, in both their spatial and temporal degrees of freedom. In the x-ray region, however, spatial control has been virtually static mainly due to stringent requirements for realizing high-performance optical elements. The lack of dynamic spatial control of x-ray beam has prevented researchers from realizing more sophisticated use of the wave field, which has rapidly advanced in the optical region in the past decades. In this study, we propose a practical scheme to dynamically control local x-ray reflectivity of a perfect silicon crystal by a femtosecond optical laser and demonstrate a programmable spatial x-ray modulator. Our modulator aims for spatial manipulation of the x-ray amplitude and is shown to produce arbitrary grayscale patterns with spatial frequencies up to 25 per millimeter. The proposed modulation scheme opens up a platform to enable advanced x-ray sensing and imaging techniques that can fully harness the wave nature of x-rays.

## Teaser

Optical laser-based spatial x-ray modulator dynamically controls x-ray beam profile at will.

## INTRODUCTION

X-rays, just like electromagnetic wave fields in the radio and optical frequencies, can encode information spatially via the amplitude, phase, and polarization of the x-ray field. Spatially resolved detections of these different channels have been used widely in scientific and industrial applications (*1*). Spatial control of x-ray fields also has had great impacts on them in recent decades. Concentric circular modulation by the Fresnel zone plates (*2*) has been established as an effective approach for nanofocusing and nanoimaging (*3*). Gratings with periodic structures enabled high-sensitivity phase-contrast imaging (*4*–*7*). X-ray beam patterned by masks has been applied for single-pixel imaging (SPI) and ghost imaging (GI) (*8*, *9*). However, all these spatial control efforts mentioned above are realized by prefabricated structures and therefore static. The level of spatial controllability at x-ray wavelength remains rather primitive, with limited studies reporting on dynamic x-ray optics, such as programmable diffraction gratings for x-rays (*10*) and extreme ultraviolet (*11*) using laser-induced phase transitions, an x-ray pulse picker with microelectromechanical systems (MEMS) (*12*), or a deformable mirror by piezoelectric devices (*13*). Dynamic optics in the optical regime, on the other hand, saw initial development rapidly evolving into mature industrial applications. Spatial degrees of freedom can be dynamically manipulated with liquid crystal on silicon-based spatial light modulators (LCOS-SLM) and digital micromirror devices (*14*, *15*). These devices have enabled advanced use of the light field in wide ranging applications, such as imaging, digital holography, optical communication, optical data processing, microstructure fabrication, optical vortex generation, adaptive optics, and femtosecond pulse shaping (*15*, *16*). Realizing a spatial x-ray modulator would immediately enable advanced x-ray imaging techniques, e.g., x-ray SPI and GI, and active/adaptive optics to correct wavefront distortion caused by x-ray optical elements and can become a cornerstone to help fully harness the wave nature of x-rays.

When considering an optical element that can precisely manipulate the electromagnetic fields, a basic expectation is the high precision of the optics at the wavelength level. This requirement makes it extremely difficult to realize optical elements for short-wavelength radiations like x-rays. Perfect single-crystal silicon is one of the very few materials which can be used for x-ray wavefront-preserving optics based on the Bragg reflection. X-rays are reflected only when the Bragg condition

$$2d{\text{sin}\theta }_{B}=\lambda$$ (1)

is satisfied (*17*). Here, *d* is the spacing of reflecting netplane, θ_B_ is the Bragg angle, and λ is the x-ray wavelength. To modulate the Bragg reflection spatially, we can change the local *d* and/or detune the local glancing angle from θ_B_ by changing the local netplane orientation, which both effectively introduce spatial distortions to the crystalline structure. At the same time, we have to keep the crystal near-perfect locally. These mutually contradicting requirements have made it extremely challenging to spatially control x-ray fields by the Bragg reflection, let alone dynamically.

In this research, we demonstrate a programmable spatial x-ray modulator driven by a femtosecond optical laser. We leverage a transient strain in a single-crystal silicon induced by the laser pulse to reflect x-rays. Using so-called back-scattering geometry, we boost the effect of subtle strain on the x-ray reflectivity, so that we can almost turn the reflection off. Furthermore, the x-ray reflectivity is spatially controlled by the laser fluence distribution on the silicon crystal. Therefore, we are able to dynamically pattern the x-ray beam by tailoring the laser beam profile with an LCOS-SLM. We discuss the optimal operation point of the modulator and evaluate the performance characteristics.

## RESULTS

### X-ray reflectivity control

As noted earlier, there are two strategies to enable local control of reflectivity, by changing the local *d* or by changing the local netplane orientation. The latter requires inclining the netplanes in neighboring small areas. This is difficult for a bulk crystal, although it might be possible with the advanced MEMS technology (*12*) in the future. In the present study, we consider controlling the local *d* instead. While x-ray reflectivity is sensitive to *d* given a fixed orientation, one recognized challenge is to achieve complete turn-off by the laser-induced strain under a common glazing incidence condition (*18*–*21*). This can be understood by differentiating Eq. 1 (see text S1 for more rigorous treatment), which describes how the lattice strain, Δ*d*/*d*, shifts θ_B_

$$\Delta \theta =-\left(∆d/d\right)\;\text{tan}\theta_{B}$$ (2)

To turn the x-ray reflection off, the magnitude of the Bragg angle shift, ∣Δθ∣, must exceed the intrinsic angular width of the Bragg reflection, Δω. If we assume tanθ_Β_ ~ 0.3 and Δω ~ 10 μrad for a typical glazing incidence condition, we need Δ*d*/*d* = 3 × 10^−5^ to achieve ∣Δθ∣ = Δω. We estimate that such a strain would require a laser fluence of about 50 mJ/cm^2^ by extrapolating our result shown later. Such high level of fluence is close to or even higher than the melting threshold of silicon around 130 mJ/cm^2^ (*22*), thus could easily damage the crystal, preventing sustained operation. Furthermore, fine patterning becomes difficult for larger strain, because a wider transient region is necessary between highly strained and strain-free regions.

Our key idea to realize a spatial x-ray modulator is making full use of the tanθ_B_ factor in Eq. 2. If we use the back-scattering geometry, i.e., θ_B_ ~ 90°, then we can make tanθ_B_ very large. In the present study, we set θ_B_ = 89.5° using the Si 555 reflection at 9.886 keV. This enhances the strain effect by a factor of tan(89.5°) = 114.6. As a result, we could induce drastic change in reflectivity at the moderate laser fluence of 1.5 mJ/cm^2^, much lower than observed laser-damage threshold. We consider that the exact back-scattering condition of θ_B_ = 90° should be avoided, because Δω also increases considerably (*17*) and overlap of the incident and reflected beams makes the experimental setup impractical. We note that Eq. 2 is valid for (∆*d*/*d*)tan^2^θ_B_ ≪ 1 and becomes inaccurate at θ_B_ = 90° (see text S1 for details).

### Spatial x-ray modulator

**Fig. 1.**
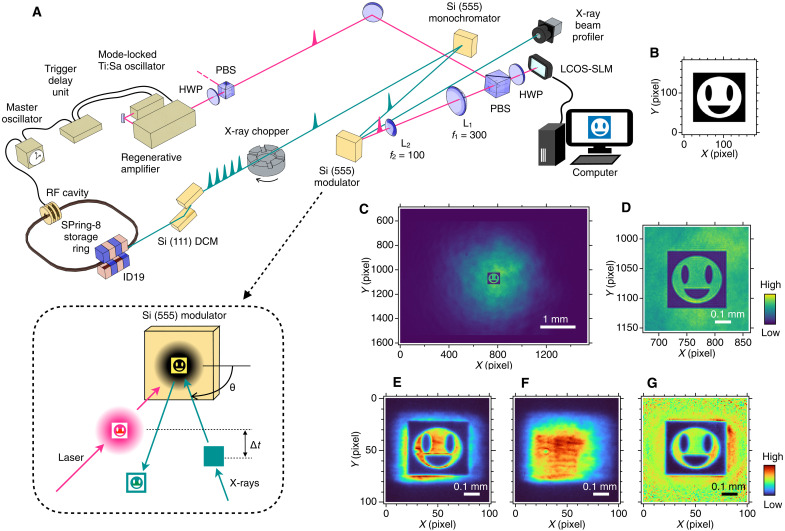
X-ray beam modulation by patterned laser illumination. (**A**) Schematic experimental setup. ID, insertion device; HWP, half-wave plate; PBS, polarizing beam splitter; L, plano-convex lens. (**B**) A binary smiley pattern input to LCOS-SLM. (**C** and **D**) Laser beam profile modulated by LCOS-SLM (C) and the magnified central region (D) measured at the modulator surface position. (**E**) X-ray beam profile modulated by the patterned laser beam shown in (C). (**F**) X-ray beam profile for an unpatterned laser beam. (**G**) X-ray beam profile (E) normalized by (F).

Figure 1A shows the schematics of our spatial x-ray modulator (see Materials and Methods for details). We used an LCOS-SLM to control the intensity profile of the 800-nm Ti:sapphire laser pulses. First, we displayed a smiley pattern on the LCOS-SLM (Fig. 1B), which modulated the laser pulse with the same pattern (Fig. 1, C and D). The smiley-patterned laser pulse created a smiley-shaped transient strain profile on the modulator surface. We aligned the modulator crystal beforehand to satisfy the Bragg condition for a particular lattice strain condition, so that only the strained region defined by the laser pattern could reflect x-rays at a particular time after the laser pulse. Last, we realized an x-ray beam patterned with the smiley pattern (Fig. 1E). We also show unpatterned x-ray beam (Fig. 1F) when the laser illumination carries no spatial modulation. The nonuniform x-ray profile was due to the upstream x-ray beamline optics and was not caused by the modulator. This serves as a reference “white field” for further imaging renormalization. The smiley profile normalized by the unpatterned beam (Fig. 1G) reproduces the input pattern well.

The modulator requires the use of one laser pulse to pattern one x-ray pulse. We used an x-ray chopper to select a single pulse from the megahertz pulse-train pattern of the SPring-8 synchrotron. The x-ray repetition rate was set to 948 Hz, matching that of the femtosecond laser (see Materials and Methods for details). The opening window of the x-ray chopper limited the beam size to a rectangular shape with 500 (*H*) × 450 (*V*) μm^2^, while the laser beam had a Gaussian profile with SD sizes of 1.0 mm (*H*) and 1.2 mm (*V*). We used only a small cross-sectional area of the laser beam to modulate the x-ray reflectivity (Fig. 1C). This reduces the variation in the total laser power absorbed by the modulator crystal when the modulation pattern was changed. This helps stabilize the crystal temperature and, hence, the operation condition.

The pattern on the LCOS-SLM is controllable via a computer and so is the resulting x-ray beam profile. As a demonstration, we took an x-ray flip video (movie S1). The LCOS-SLM patterns (fig. S1) were proceeded one by one at 1 Hz, while the x-ray beam profile was monitored with an exposure time of 0.5 s. Some frames include transition from one pattern to the next, because the profile was captured in an asynchronous manner. Here, we emphasize that the flip video is a live-view movie obtained during the modulator operation. It does not require any image reconstruction process from a series of still images taken separately. The speed of the pattern transition would only be limited by the response time of the LCOS-SLM, which is about 200 ms for the present system. On the other hand, the modulator reflectivity changes on the order of nanosecond, as we explain in detail next.

### Determination of operation point

**Fig. 2.**
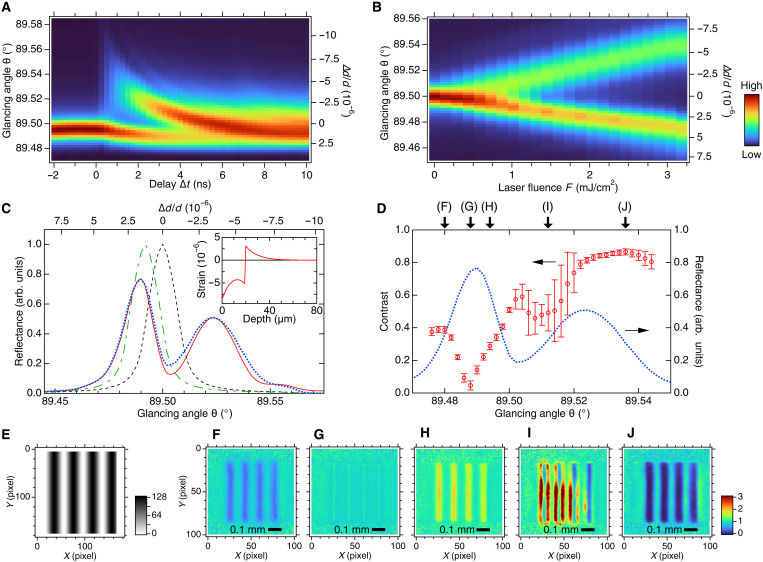
Determination of operating condition. (**A** and **B**) Rocking curves of the modulator Si 555 reflection as a function of Δ*t* for *F* = 1.5 mJ/cm^2^ (A) and a function of *F* for Δ*t* = 2ns (B). Positive delays represent x-ray reflection after the laser illumination (A). (**C**) Rocking curves of the modulator under a laser-off condition (dashed line), at Δ*t* = −2 ns (dot-dashed line) and 2 ns (dotted line). Solid line represents numerically simulated curve for Δ*t* = 2 ns using a calculated strain profile shown in inset (see text for details). (**D**) Open circles indicate fringe contrast of x-ray beam measured at Δ*t* = 2 ns. Vertical bars are the SD. Dotted line represents the rocking curve at Δ*t* = 2 ns reproduced from (C). (**E**) A grayscale sinusoidal-fringe pattern input to the LCOS-SLM. The spatial frequency was set 1/40 pixel^−1^, which corresponded 9.13 mm^−1^ on the modulator surface. (**F** to **J**) Normalized x-ray beam profiles measured for the fringe pattern (E) at characteristic glancing angles, 89.480° (F), 89.488° (G), 89.494° (H), 89.512° (I), and 89.536° (J), which are indicated by arrows in (D).

Operation condition of the modulator is essential to obtain a clear x-ray beam profile. We have to consider multiple parameters, namely, the laser fluence, *F*, the laser-x-ray time delay, Δ*t*, and the glancing angle of x-rays, θ. Optimization of these parameters is not straightforward, because they are interconnected with each other. We start with Δ*t*. Figure 2A shows the Δ*t* dependence of the rocking curve. Note that a rocking curve represents x-ray reflectance as a function of θ. The average laser power and *F* on the modulator surface were 120 mW and 1.5 mJ/cm^2^, respectively. Just after the laser excitation, the Bragg peak shifted slightly toward the lower θ side, and a new branch emerged in the high θ region. Higher θ corresponds to negative strain as can be understood from Eq. 2. Thus, the new branch originates from a compressed region of the lattice. We refer to this as an upper branch. On the other hand, the lower branch comes from the dilated region of the lattice. The condition of ∣Δθ∣ > Δω can be fulfilled for the upper branch within a narrow positive range in Δ*t*.

When choosing Δ*t*, we needed to consider two counteracting factors. One is the angular shift of the upper branch, which relates to the contrast of the x-ray beam pattern. The contrast is given by (*R_F_* − *R*_0_)/(*R_F_* + *R*_0_). Here, *R_F_* is the reflectivity achieved at maximum local *F*, and *R*_0_ is the reflectivity at locations where *F* = 0. We can also evaluate *R*_0_ from the reflectivity at the same angle but for Δ*t* < 0 in Fig. 2A. Higher contrast requires lower *R*_0_ and, therefore, is achievable in the region where the angular shift of the upper branch is large. The other factor is the absolute x-ray reflectivity, i.e., *R_F_*, which determines the modulator efficiency as well as the contrast. The angular shift of the upper branch is larger at smaller Δ*t*, whereas *R_F_* becomes higher at lager Δ*t*. In the present study, we chose Δ*t* = 2 ns, considering the efficiency.

We next consider the fluence parameter, *F*. Figure 2B shows the *F* dependence of the rocking curve at Δ*t* = 2 ns. The angular shifts of both branches are found to be almost linear with respect to *F*. The large angular shift at high *F* could improve the contrast. However, the modulator may become unstable thermally at high *F* for the current setup without active cooling. We observed thermal drift at the highest *F*, where the laser power on the modulator surface averaged 240 mW. As a result, we chose *F* = 1.5 mJ/cm^2^ as a sweet spot compromising among contrast, efficiency, and stability. We could realize enough imaging contrast under this condition as we will see later. Active control of the modulator crystal temperature would allow us to use higher *F* for the better contrast. We note that *F* in some previously reported time-resolved x-ray diffraction experiments was much higher (*18*–*21*); nevertheless, considerable reflection efficiency was observed in those experiments.

Figure 2C shows the rocking curves measured at Δ*t* = −2 and 2 ns with *F* = 1.5 mJ/cm^2^, together with that under a laser-off condition. The angular difference between the laser-off condition and Δ*t* = −2 ns is only 0.006°, which corresponds to a lattice constant difference of δ*d*/*d* = 9.2 × 10^−7^. The modulator crystal was considered to be in a thermal equilibrium state at Δ*t* = −2 ns, i.e., after about 1 ms from the last laser excitation. We estimate the overall average crystal temperature rise due to the laser to be only (δ*d*/*d*)/β = 0.35 K, where β = 2.59 × 10^−6^ K^−1^ is the linear expansion coefficient of silicon (table S1). This temperature increase was sufficiently small to enable stable operation without cooling.

To better understand how the two factors, Δ*t* and *F*, determine the modulator response, we performed numerical simulations of the ultrafast response of a silicon crystal under short-pulse laser illumination following what was described in (*23*) (see Materials and Methods for details). The laser pulse simultaneously excites many electrons from the valence band to the conduction band. The dense electron-hole pairs induce an electronic stress, which is negative for silicon. The hot electrons relax quickly in about 1 ps to the bottom of the conduction band, giving thermal energy to the lattice. The negative electronic stress is stronger than the positive thermal one for silicon and generates a dilation-leading compression bipolar strain pulse, i.e., a shock wave, traveling inward at a sound velocity. One can calculate the strain profile for given Δ*t* and *F* (Fig. 2C, inset) based on the analytical Thomsen model (*23*, *24*). On a much longer timescale of several 100 ns and beyond, the electron-hole pairs recombine and release additional thermal energy, which diffuses over the crystal.

Once the strain profile is given, the rocking curve can be calculated using the Takagi-Taupin theory (*25*, *26*). We adjusted Δ*t* and *F*, so that the theoretical curve reproduced the measured rocking curve at Δ*t* = 2 ns well, and found good agreement for Δ*t* = 2.1 ns and *F* = 3.33 mJ/cm^2^. The time difference of 0.1 ns between the simulation and experiment is within the uncertainty of the time-zero determination (see Materials and Methods and fig. S2), while *F* is 2.22 times higher than the experimental condition. We consider that the discrepancy in *F* is due to the underestimated strain in the Thomsen model, which treats the energy transfer to the lattice as instantaneous and ignores diffusion (*27*). The reflectivity of the upper branch in Fig. 2A is determined by thickness of the compressed layer, which is about 20 μm at Δ*t* = 2.1 ns (Fig. 2C, inset). As the shock wave propagates inward, the compressed layer becomes thicker, increasing the reflectivity. At the same time, the angular shift of the upper branch decreases because of the lower averaged strain. The reflectivity of the lower branch decreases, because the dilated region moves deeper into the crystal as the shock wave propagates and eventually beyond the x-ray penetration depth. This picture explains Fig. 2A well.

We lastly consider the glancing angle parameter, θ. While one natural choice of operation angle is the peak of the upper branch, we chose a θ that achieved the highest contrast. Figure 2D shows the θ dependence of the contrast for vertical sinusoidal fringes (Fig. 2E) with a spatial frequency of ξ = 9.13 mm^−1^. We evaluated the contrast for the fringe profile normalized by the unpatterned beam taken at each θ (Fig. 2, F to J). The highest contrast is 0.87 obtained at θ = 89.536° (Fig. 2J), slightly higher angle than the Bragg peak at 89.524°. This is because the falloff of the reflectivity for *F* = 0 is faster than that for the maximum *F* until 89.536°. This θ dependence of the reflectivity improves the contrast even when θ is detuned from the Bragg peak.

Large errors of the contrast between θ = 89.498° and 89.514° are due to extra fringes as shown in Fig. 2I. In this angular range, x-ray reflectivity is not monotonic with respect to *F* and decreases above a certain *F*, which inserts an extra fringe between the neighboring main fringes. The x-ray beam image shows reversed contrast around θ = 89.49° (Fig. 2H), where the *F* dependence is opposite, i.e., lower x-ray reflectivity at higher *F*. The x-ray profile (Fig. 2F) is most faithful to the input at θ = 89.478°, the edge of the lower branch (Fig. 2D), but the contrast of only 0.39 may be undesirable for practical applications.

### Basic performance of spatial x-ray modulator

**Fig. 3.**
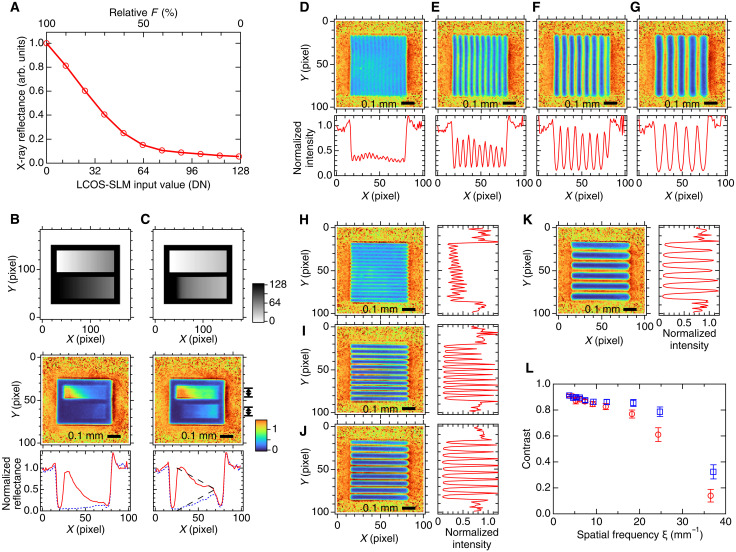
Performance of spatial x-ray modulator. (**A**) Local x-ray reflectance as a function of the LCOS-SLM input value in digital number (DN), which is proportional to the reduction of *F*. (**B** and **C**) X-ray beam profiles for a linear gradation (B) and a linearity-calibrated gradation input patterns (C). Top shows the grayscale input patterns to the LCOS-SLM. Middle is the normalized x-ray beam profiles. Bottom represents horizontal line profiles averaged vertically. The width over which the profile is averaged is indicated in the middle-right. Dashed lines in the lower-right are guide for the eye. (**D** to **G**) Normalized x-ray images (top) and section profiles along *Y* = 50 (bottom) for vertical fringes with spatial frequencies, ξ = 36.5 mm^−1^ (D), 24.4 mm^−1^ (E), 18.3 mm^−1^ (F), and 12.2 mm^−1^ (G). (**H** to **K**) Normalized x-ray images (left) and section profiles along *X* = 50 (right) for horizontal fringes with spatial frequencies, ξ = 37.1 mm^−1^ (H), 24.8 mm^−1^ (I), 18.6 mm^−1^ (J), and 12.4 mm^-1^ (K). (**L**) Contrast of horizontal (circles) and vertical (squares) fringe patterns as a function of the spatial frequency. Vertical bars indicate the SD.

We evaluate in this section the modulator performance quantitatively at the operation point of Δ*t* = 2 ns, *F* = 1.5 mJ/cm^2^, and θ = 89.536°. First, we discuss the modulator sensitivity to *F*. The *F* dependence of the reflectivity at a fixed θ is not linear, because it should have a similar shape to the rocking curve as is understood from Fig. 2B. Figure 3A shows the x-ray reflectance of the modulator at the operation point as a function of the input value to the LCOS-SLM, which is proportional to the reduction of *F*. The x-ray reflectivity quickly drops by about 80%, when *F* is reduced by only 40%. As a result, the modulator does not reproduce grayscale input patterns well. For example, an input pattern with linear gradation did not produce an x-ray linear gradient pattern as shown in Fig. 3B. However, we could make use of the measured *F* dependence (Fig. 3A) to calibrate the modulator response. Figure 3C shows x-ray beam profile taken for a linearity-calibrated input pattern. The cross-sectional profiles using the calibrated input exhibit nearly linear gradations as intended.

The spatial resolution is another important performance metric, which we evaluated on the basis of the contrast of x-ray fringe patterns with various spatial frequencies (Fig. 3, D to K). In this evaluation, we adapted the linearity calibration to the LCOS-SLM input to produce sinusoidal x-ray fringes. The contrast of the fringe pattern is calculated for the normalized image and is plotted in Fig. 3L. The contrast remains sufficiently high (>40%) up to ξ = 25 mm^−1^, which corresponds to a period of 40 μm. We may be able to interpret Fig. 3L as a modulation transfer function of the whole system, including the computer to which we input a sinusoidal fringe pattern, the LCOS-SLM which displays a linearity-calibrated pattern, and the modulator crystal lastly patterning the x-ray beam.

The deviation of the x-ray beam profile from the LCOS-SLM input pattern is not only due to the nonlinear response to *F*. The x-ray gradation patterns (Fig. 3, B and C) are not uniform vertically especially in the left-side regions of the upper gradation bars. This implies that the local x-ray reflectivity at a specific position can be influenced by the laser fluence in the surrounding area. One may also notice distortion of the vertical fringe patterns (Fig. 3, D to G). We consider that the distortion is not intrinsic but the result of misalignment between the two lenses (L_1_ and L_2_ in Fig. 1A).

The x-ray fringe pattern is visible beyond ξ = 35 mm^−1^ (Fig. 3, D and H). However, the spatial resolution is poorer than that expected from limitations by optics. The demagnified pixel size of the LCOS-SLM on the modulator surface was 2.74 (*H*) × 2.69 (*V*) μm^2^ and the pixel size of the x-ray beam monitor was 6.5 μm. We speculate that the spatial resolution is limited by the modulator response, i.e., lateral blur of the laser induced strain. When the laser profile is not uniform, the strain pulse can propagate not only along the depth direction but also along the surface. The contrast is found to be higher for the horizontal fringe than that for the vertical fringe, which may be due to difference in the sound velocity between the two directions.

## DISCUSSION

In summary, we have realized a programmable spatial x-ray modulator, which can produce arbitrary grayscale x-ray patterns with spatial frequencies up to 25 mm^−1^. The present modulator was operated at a repetition rate of 948 Hz, which considerably reduced the available photon flux of the patterned beam. An active temperature control of the modulator crystal would allow higher repetition rate, e.g., 10 kHz and 10-fold increase in the photon flux. All pulses can be patterned for low-repetition rate x-ray free-electron lasers (*28*–*31*), which are equipped with synchronized Ti:sapphire laser systems. There, the photon flux is reduced only by the photon energy resolution of the modulator crystal. The total number of pixels within the field of view may in principle be limited by the required fluence of 1.5 mJ/cm^2^. For example, if we match the effective pixel size of the modulator to the observed spatial resolution of 40 μm and assume a relatively high pulse energy of 5 mJ, which might require an elaborate cooling of the modulator, then the maximum pixel number and the largest field of view could be 0.2 Mpixels and 320 mm^2^, respectively.

We briefly discuss one straightforward application of the present spatial x-ray modulator. With patterned beam illumination, x-ray SPI and GI can image a sample using signals taken by a bucket detector without spatial resolution, overcoming the limitations of imaging detectors (*8*, *9*, *14*, *32*–*40*). The contrast ratio of the image obtained by SPI and GI is better than the normal imaging under a low signal-to-noise ratio condition (*41*). The importance of the spatial x-ray modulator originates from the fact that it can easily optimize the illumination pattern to each sample, making the measurement more dose efficient (*14*, *42*). Since x-rays are harmful to biological systems, less-damaging x-ray SPI and GI with the optimized illumination would be advantageous for investigating radiation-sensitive specimens (*36*). For low-dose applications, the relatively low throughput of the present spatial x-ray modulator would still provide sufficient flux. Furthermore, the spatial resolution of SPI and GI is determined by the pixel size of the modulator, i.e., 2.8 μm in the present study, rather than the patterning resolution of 40 μm. This is because the x-ray beam pattern can be shifted by a step size as small as the pixel size of the modulator.

Last, we note that the present spatial x-ray modulator can also control the local x-ray phase as well, because the phase shift at the reflection depends on the deviation from the Bragg condition (*17*). When the incurred phase shift is small, change in the reflectivity will be negligible. The local phase control can thus be used for active optics to correct small fabrication errors of x-ray optical elements, such as mirrors and lenses, and for adaptive optics by virtue of the programmable patterning.

## MATERIALS AND METHODS

### Experimental setup

The experiments were performed at BL19LXU of SPring-8 (*43*). The x-ray beam from a 27-m in-vacuum undulator was monochromatized to 9.886 keV by a cryogenically cooled Si (111) double-crystal monochromator (DCM). The photon energy was selected to make the Bragg angle of the Si 555 reflection to be 89.5°. The DCM bandwidth of 1.3 eV was too broad to operate the modulator. Therefore, we further monochromatized the x-ray beam using the same Si 555 reflection as the modulator (Fig. 1A). Then, a 15-meV beam was delivered to the modulator, which was a silicon (111) single-crystal plate with a thickness of 3 mm. The output beam image of the modulator was measured by an x-ray beam profiler with a pixel size of 6.5 μm (*44*).

The storage ring was operated in a hybrid bunch mode (H mode), where a 5-mA single electron bunch is isolated by 1.487 μs from a remaining bunch train containing a current of 95 mA. An x-ray chopper (*45*) was inserted after the DCM to select a 40-ps x-ray pulse generated from this single electron bunch every 220 revolutions, i.e., at 948 Hz. The aperture of the x-ray chopper limited the x-ray beam size to 500 (*H*) × 450 (*V*) μm^2^. A Ti:sapphire laser system, which consisted of a mode-locked oscillator (Tsunami, Spectra-Physics) and a regenerative amplifier (Spitfire Ace, Spectra-Physics), produced 800-nm, 120-fs, and 0.6-mJ pulses at the same repetition rate as the x-ray pulse. The maximum laser power used was 240 mW on the modulator surface. The laser system was synchronized to the radio-frequency signal of the storage ring with an accuracy of 4 ps (*46*). The relative time delay between the x-ray and laser pulses was controlled electronically. The time zero was determined by onset of the reflectivity change with an accuracy of 100 ps (fig. S2).

We used an LCOS-SLM (SLM-200, Santec) in an amplitude mode to spatially modulate the laser pulse profile with arbitrary patterns on the modulator. A polarizing beam splitter and a half-wave plate converted the phase pattern displayed on the LCOS-SLM to the intensity distribution of the beam cross section (Fig. 1A). The LCOS-SLM pattern was transferred to the surface of the modulator crystal by a 4*f* optical system, which consisted of two plane-convex lenses with *f* = 300 and 100 mm and demagnified the image by a factor of 3. To avoid x-rays hitting the optical lens, the incident angle of the laser beam was tilted by 11.2° from the surface normal in the horizontal plane. We checked the laser beam profile at the modulator position using a beam profiler with a pixel size of 3.45 μm (SP920s, Ophir), which could be swapped for the modulator by a motorized stage. Using the beam profiler, the actual demagnification ratios were measured to be 2.92 (*H*) and 2.97 (*V*). The pixel pitch of the LCOS-SLM was 8.0 μm; therefore, the nominal pixel size of the modulator was 2.8 (*H*) × 2.7 (*V*) μm^2^. The pixel number of the LCOS-SLM was 1920 (*H*) × 1200 (*V*).

### Takagi-Taupin simulation

The numerical simulation consists of three steps. The first step is to calculate the strain profile of the modulator crystal along the depth direction for the given Δ*t* and *F*. We use the Thomsen model (*23*, *24*), where propagation of the strain pulse is described by an analytical solution of the equations of elasticity. Then, we calculate the rocking curve of the strained Si (555) crystal using the Takagi-Taupin theory (*25*, *26*). We take the layer approximation (*47*) and divide the 80-μm-thick region into 1-μm-thick lamellae. Within each lamella, we assume a constant strain given by its central value. The calculation is performed by using the formula given in the previous report (*47*) except for the parameter

$$\alpha =4\text{sin}\theta_{B}\left\{\text{sin}\theta_{B}-\left(1+\frac{\Delta d}{d}\right)\text{sin}\theta \right\}$$ (3)

which is more accurate for the present back-scattering geometry. Last, we calculate the convolution between the rocking curves for the modulator and the Si (555) monochromator to take its energy spread into account. The physical quantities used in the simulation are taken from literatures, which are summarized in table S1.

## Supplementary Materials

### The PDF file includes:

Supplementary Text S1 Figs. S1 and S2 Table S1 Legend for movie S1 References
