# Supplementary material for: Dynamically patterning x-ray beam by a femtosecond optical laser
Source: Sci Adv. 2024 Nov 20;10(47):eadp5326. doi: 10.1126/sciadv.adp5326 (PMC11578175; doi:10.1126/sciadv.adp5326)
Supplement: Supplementary file 1 — Supplementary Text S1 Figs. S1 and S2 Table S1 Legend for movie S1 References [file sciadv.adp5326_sm.pdf]

Science Advances
AAAS

Supplementary Materials for

Dynamically patterning x-ray beam by a femtosecond optical laser

Kenji Tamasaku *et al.*

Corresponding author: Kenji Tamasaku, tamasaku@riken.jp

Sci. Adv. **10**, eadp5326 (2024)
DOI: 10.1126/sciadv.adp5326

The PDF file includes:

Supplementary Text S1
Figs. S1 and S2
Table S1
Legend for movie S1
References

Other Supplementary Material for this manuscript includes the following:

Movie S1

Supplementary Text

Text S1. Shift of Bragg angle by lattice strain

The relational expression Eq. (2) between the shift of Bragg angle, $\Delta\theta$, and the lattice strain, $\Delta d/d$, is not applicable to θ_B=90° because of the divergence of $\tan\theta_B$. Here, we discuss a more rigorous treatment, but within the kinematical framework.

Introducing $\Delta\theta$ and Δd into Eq. (1), we obtain:

$$2(d + \Delta d)\sin(\theta_B + \Delta\theta) = \lambda\,. \tag{S1}$$

This equation can be solved analytically:

$$\Delta\theta = \sin^{-1}\left(\frac{\sin\theta_B}{1+\Delta d/d}\right) - \theta_B. \tag{S2}$$

Although Eq. (S2) is the exact solution, it is difficult to understand the $\Delta d/d$ dependence of $\Delta\theta$. Below we limit our discussion to a weak strain condition, i.e., $\Delta d \ll d$. In this case, Eq. (S1) can be rewritten as a quadratic equation of $\Delta\theta$. Since $\Delta\theta \ll 1$, it is possible to replace $\sin\Delta\theta$ by $\Delta\theta$ and $\cos\Delta\theta$ by $1 - \Delta\theta^2/2$. The solution to the quadratic equation may be given by:

$$\Delta\theta \simeq \frac{1-\sqrt{1+2(\Delta d/d)\tan^2\theta_B}}{\tan\theta_B}. \tag{S3}$$

This formula can be simplified when $(\Delta d/d)\tan^2\theta_B \ll 1$:

$$\Delta\theta \simeq -(\Delta d/d)\tan\theta_B. \tag{S4}$$

This expression is identical to Eq. (2).

In the case of the exact back-scattering geometry, i.e., θ_B=90°, the condition, $(\Delta d/d)\tan^2\theta_B \ll 1$, is no longer satisfied. In the limit of $\tan\theta_B$ approaching infinity, Eq. (S3) can be reduced to:

$$\Delta\theta \simeq -\sqrt{2\Delta d/d}. \tag{S5}$$

Note that Eq. (S5) can also be derived directly from Eq. (S2) by setting θ_B=90° and using $\sin^{-1}(1-x) \simeq \pi/2 - \sqrt{2x}$ for $x \ll 1$.

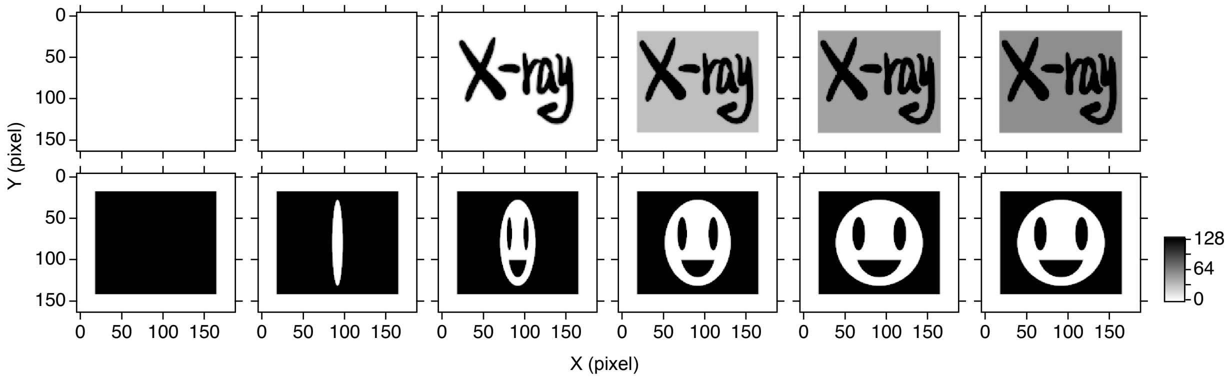

Fig. S1. Grayscale Input patterns to LCOS-SLM for Movie S1.

The actual input data has a pixel number of 1920(H)×1200(V), but only the central region including a pattern is shown. Low input values to the LCOS-SLM yielded high *F*, making the X-ray reflectivity high.

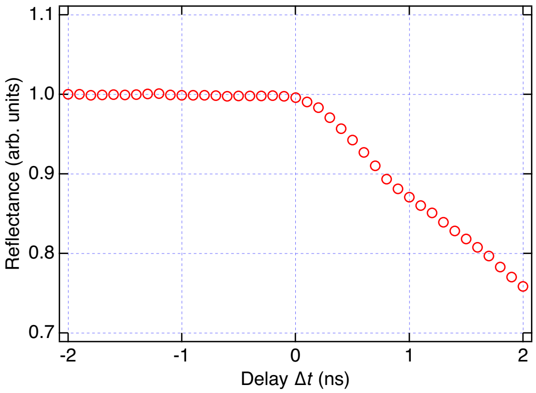

Fig. S2. Determination of time zero.

Delay scan of the X-ray reflectance on the Bragg peak at Δt=-2 ns. The time zero was determined as the onset of decrease in the reflectance.

Table S1. Physical quantities used in the Takagi-Taupin simulation.
The sound velocity was calculated by $v_{111}=\{(C_{11}+2C_{12}+4C_{44})/3\rho\}^{1/2}$.

Physical quantity	Symbol	Value	Reference
Elastic constant	C_{11}	1.6577×10^{11} Pa	(*48*)
	C_{12}	0.6393×10^{11} Pa	(*48*)
	C_{44}	0.7962×10^{11} Pa	(*48*)
Sound velocity along the [111] direction	v_{111}	9.36×10^{3} m/s	
Bulk modulus	B	9.783×10^{10} Pa	(*48*)
Specific heat	C	1.66×10^{6} J/m^{3}K	(*48*)
Linear expansion coefficient	β	2.59×10^{-6} 1/K	(*48*)
Density	ρ	2.329×10^{3} Kg/m^3	(*48*)
Band gap	E_g	1.12 eV	(*48*)
	$\partial E_g/\partial p$	-1.41×10^{-11} eV/Pa	(*48*)
Reflectivity at 800 nm	R	0.3294	(*49*)
Penetration depth at 800 nm	$1/\alpha$	9.729×10^{-6} m	(*49*)
Fourier coefficient of electric susceptibility at 9.886 keV	χ_0	$-9.9913\times10^{-6}-1.5560\times10^{-7}i$	(*50*)
	χ_{555}	$-1.4502\times10^{-6}-8.1073\times10^{-8}i$	(*50*)

Movie S1. X-ray flip video.

This movie shows the live-view screen of the X-ray beam monitor. The LCOS-SLM displayed the patterns shown in fig. S1 in a sequential manner at 1 Hz.

REFERENCES AND NOTES

1. J. Als-Nielsen, D. McMorrow, *Elements of Modern X-ray Physics* (Wiley, 2011).

2. J. Kirz, Phase zone plates for x rays and the extreme uv. *J. Opt. Soc. Am.* **64**, 301–309 (1974).

3. A. Sakdinawat, D. Attwood, Nanoscale x-ray imaging. *Nat. Photonics* **4**, 840–848 (2010).

4. C. David, B. Nöhammer, H. H. Solak, E. Ziegler, Differential x-ray phase contrast imaging using a shearing interferometer. *Appl. Phys. Lett.* **81**, 3287–3289 (2002).

5. A. Momose, S. Kawamoto, I. Koyama, Y. Hamaishi, K. Takai, Y. Suzuki, Demonstration of x-ray talbot interferometry. *Jpn. J. Appl. Phys.* **42**, L866–L868 (2003).

6. F. Pfeiffer, T. Weitkamp, O. Bunk, C. David, Phase retrieval and differential phase-contrast imaging with low-brilliance x-ray sources. *Nat. Phys.* **2**, 258–261 (2006).

7. H. Miao, A. Panna, A. A. Gomella, E. E. Bennett, S. Znati, L. Chen, H. Wen, A universal moiré effect and application in x-ray phase-contrast imaging. *Nat. Phys.* **12**, 830–834 (2016).

8. Y.-H. He, A.-X. Zhang, M.-F. Li, Y.-Y. Huang, B.-G. Quan, D.-Z. Li, L.-A. Wu, L.-M. Chen, High-resolution sub-sampling incoherent x-ray imaging with a single-pixel detector. *APL Photon.* **5**, 056102 (2020).

9. A. Aminzadeh, L. Roberts, B. Young, C.-I. Chiang, I. D. Svalbe, D. M. Paganin, A. M. Kingston, Mask design, fabrication, and experimental ghost imaging applications for patterned x-ray illumination. *Opt. Express* **31**, 24328–24346 (2023).

10. J. Park, P. Zalden, E. Ng, S. Johnston, S. Fong, C. Chang, C. Tassone, D. Van Campen, W. Mok, H. Mabuchi, H. Wong, Z. Shen, A. Lindenberg, and A. Sakdinawat, Laser-induced patterning for a diffraction grating using the phase change material of $Ge_2Sb_2Te_5$ (GST) as a spatial light modulator in x-ray optics: A proof of concept. *Opt. Mater. Express* **12**, 1408–1416 (2022).

11. I. Vaskivskyi, A. Mraz, R. Venturini, G. Jecl, Y. Vaskivski, R. Mincigrucci, L. Foglia, D. De Angelis, J.-S. Pelli-Cresi, E. Paltanin, D. Fainozzi, F. Bencivenga, C. Masciovecchio, D. Mihailovic, A high-

efficiency programmable modulator for extreme ultraviolet light with nanometre feature size based on an electronic phase transition. *Nat. Photonics* **18**, 458–463 (2024).

12. P. Chen, I. Jung, D. Walko, Z. Li, Y. Gao, T. Mooney, G. Shenoy, D. Lopez, and J. Wang, Optics-on-a-chip for ultrafast manipulation of 350-MHz hard x-ray pulses. *Opt. Express* **29**, 13624–13640 (2021).

13. S. G. Alcock, I.-T. Nistea, V. G. Badami, R. Signorato, K. Sawhney, High-speed adaptive optics using bimorph deformable x-ray mirrors. *Rev. Sci. Instrum.* **90**, 021712 (2019).

14. G. M. Gibson, D. Johnson, M. J. Padgett, Single-pixel imaging 12 years on: A review. *Opt. Express* **28**, 28190–28208 (2020).

15. U. Efron, in *Handbook of Optoelectronics Volume II*, J. P. Dakin, R. G. W. Brown, Eds. (CRC Press, 2017), pp. 225–268.

16. G. Lazarev, P.-J. Chen, J. Strauss, N. Fontaine, A. Forbes, Beyond the display: Phase-only liquid crystal on Silicon devices and their applications in photonics. *Opt. Express* **27**, 16206–16249 (2019).

17. A. Authier, *Dynamical Theory of X-Ray Diffraction* (Oxford Univ. Press, 2001).

18. C. Rose-Petruck, R. Jimenez, T. Guo, A. Cavalleri, C. W. Siders, F. Rksi, J. A. Squier, B. C. Walker, K. R. Wilson, C. P. J. Barty, Picosecond-milliångström lattice dynamics measured by ultrafast x-ray diffraction. *Nature* **398**, 310–312 (1999).

19. A. M. Lindenberg, I. Kang, S. L. Johnson, T. Missalla, P. A. Heimann, Z. Chang, J. Larsson, P. H. Bucksbaum, H. C. Kapteyn, H. A. Padmore, R. W. Lee, J. S. Wark, R. W. Falcone, Time-resolved x-ray diffraction from coherent phonons during a laser-induced phase transition. *Phys. Rev. Lett.* **84**, 111–114 (2000).

20. D. A. Reis, M. F. DeCamp, P. H. Bucksbaum, R. Clarke, E. Dufresne, M. Hertlein, R. Merlin, R. Falcone, H. Kapteyn, M. M. Murnane, J. Larsson, Th. Missalla, J. S. Wark, Probing impulsive strain propagation with x-ray pulses. *Phys. Rev. Lett.* **86**, 3072–3075 (2001).

21. Y. Hayashi, Y. Tanaka, T. Kirimura, N. Tsukuda, E. Kuramoto, T. Ishikawa, Acoustic pulse echoes probed with time-resolved x-ray triple-crystal diffractometry. *Phys. Rev. Lett.* **96**, 115505 (2006).

22. C. Florian, D. Fischer, K. Freiberg, M. Duwe, M. Sahre, S. Schneider, A. Hertwig, J. Krüger, M. Rettenmayr, U. Beck, A. Undisz, J. Bonse, Single femtosecond laser-pulse-induced superficial amorphization and re-crystallization of silicon. *Materials* **14**, 1651 (2021).

23. O. B. Wright, V. E. Gusev, Acoustic generation in crystalline silicon with femtosecond optical pulses. *Appl. Phys. Lett.* **66**, 1190–1192 (1995).

24. C. Thomsen, H. T. Grahn, H. J. Maris, J. Tauc, Surface generation and detection of photons by picosecond light pulses. *Phys. Rev. B* **34**, 4129–4138 (1986).

25. S. Takagi, A dynamical theory of diffraction for a distorted crystal. *J. Phys. Soc. Jpn.* **26**, 1239–1253 (1969).

26. D. Taupin, Théorie dynamique de la diffraction des rayon X par les cristaux déformés. *Bull. Soc. Fr. Minér. Crist.* **87**, 469–511 (1964).

27. B. Lings, J. S. Wark, M. F. DeCamp, D. A. Reis, S. Fahy, Simulations of time-resolved x-ray diffraction in Laue geometry. *J. Phys. Condens. Matter* **18**, 9231–9244 (2006).

28. P. Emma, R. Akre, J. Arthur, R. Bionta, C. Bostedt, J. Bozek, A. Brachmann, P. Bucksbaum, R. Coffee, F.-J. Decker, Y. Ding, D. Dowell, S. Edstrom, A. Fisher, J. Frisch, S. Gilevich, J. Hastings, G. Hays, Ph. Hering, Z. Huang, R. Iverson, H. Loos, M. Messerschmidt, A. Miahnahri, S. Moeller, H.-D. Nuhn, G. Pile, D. Ratner, J. Rzepiela, D. Schultz, T. Smith, P. Stefan, H. Tompkins, J. Turner, J. Welch, W. White, J. Wu, G. Yocky, J. Galayda, First lasing and operation of an ångstrom-wavelength free-electron laser. *Nat. Photonics* **4**, 641–647 (2010).

29. T. Ishikawa, H. Aoyagi, T. Asaka, Y. Asano, N. Azumi, T. Bizen, H. Ego, K. Fukami, T. Fukui, Y. Furukawa, S. Goto, H. Hanaki, T. Hara, T. Hasegawa, T. Hatsui, A. Higashiya, T. Hirono, N. Hosoda, M. Ishii, T. Inagaki, Y. Inubushi, T. Itoga, Y. Joti, M. Kago, T. Kameshima, H. Kimura, Y. Kirihara, A. Kiyomichi, T. Kobayashi, C. Kondo, T. Kudo, H. Maesaka, X. M. Maréchal, T. Masuda, S. Matsubara, T. Matsumoto, T. Matsushita, S. Matsui, M. Nagasono, N. Nariyama, H. Ohashi, T. Ohata, T. Ohshima,

S. Ono, Y. Otake, C. Saji, T. Sakurai, T. Sato, K. Sawada, T. Seike, K. Shirasawa, T. Sugimoto, S. Suzuki, S. Takahashi, H. Takebe, K. Takeshita, K. Tamasaku, H. Tanaka, R. Tanaka, T. Tanaka, T. Togashi, K. Togawa, A. Tokuhisa, H. Tomizawa, K. Tono, S. Wu, M. Yabashi, M. Yamaga, A. Yamashita, K. Yanagida, C. Zhang, T. Shintake, H. Kitamura, N. Kumagai, A compact x-ray free-electron laser emitting in the sub-ångström region. *Nat. Photonics* **6**, 540–544 (2012).

30. H.-S. Kang, C.-K. Min, H. Heo, C. Kim, H. Yang, G. Kim, I. Nam, S. Y. Baek, H.-J. Choi, G. Mun, B. R. Park, Y. J. Suh, D. C. Shin, J. Hu, J. Hong, S. Jung, S.-H. Kim, K.-H. Kim, D. Na, S. S. Park, Y. J. Park, J.-H. Han, Y. G. Jung, S. H. Jeong, H. G. Lee, S. Lee, S. Lee, W.-W. Lee, B. Oh, H. S. Suh, Y. W. Parc, S.-J. Park, M. H. Kim, N.-S. Jung, Y.-C. Kim, M.-S. Lee, B.-H. Lee, C.-W. Sung, I.-S. Mok, J.-M. Yang, C.-S. Lee, H. Shin, J. H. Kim, Y. Kim, J. H. Lee, S.-Y. Park, J. Kim, J. Park, I. Eom, S. Rah, S. Kim, K. H. Nam, J. Park, J. Park, S. Kim, S. Kwon, S. H. Park, K. S. Kim, H. Hyun, S. N. Kim, S. Kim, S.-M. Hwang, M. J. Kim, C.-Y. Lim, C.-J. Yu, B.-S. Kim, T.-H. Kang, K.-W. Kim, S.-H. Kim, H.-S. Lee, H.-S. Lee, K.-H. Park, T.-Y. Koo, D.-E. Kim, I. S. Ko, Hard x-ray free-electron laser with femtosecond-scale timing jitter. *Nat. Photonics* **11**, 708–713 (2017).

31. C. J. Milne, T. Schietinger, M. Aiba, A. Alarcon, J. Alex, A. Anghel, V. Arsov, C. Beard, P. Beaud, S. Bettoni, M. Bopp, H. Brands, M. Brönnimann, I. Brunnenkant, M. Calvi, A. Citterio, P. Craievich, M. C. Divall, M. Dällenbach, M. D’Amico, A. Dax, Y. Deng, A. Dietrich, R. Dinapoli, E. Divall, S. Dordevic, S. Ebner, C. Erny, H. Fitze, U. Flechsig, R. Follath, F. Frei, F. Gärtner, R. Ganter, T. Garvey, Z. Geng, I. Gorgisyan, C. Gough, A. Hauff, C. P. Hauri, N. Hiller, T. Humar, S. Hunziker, G. Ingold, R. Ischebeck, M. Janousch, P. Juranić, M. Jurcevic, M. Kaiser, B. Kalantari, R. Kalt, B. Keil, C. Kittel, G. Knopp, W. Koprek, H. T. Lemke, T. Lippuner, D. L. Sancho, F. Löhl, C. Lopez-Cuenca, F. Märki, F. Marcellini, G. Marinkovic, I. Martiel, R. Menzel, A. Mozzanica, K. Nass, G. L. Orlandi, C. O. Loch, E. Panepucci, M. Paraliev, B. Patterson, B. Pedrini, M. Pedrozzi, P. Pollet, C. Pradervand, E. Prat, P. Radi, J.-Y. Raguin, S. Redford, J. Rehanek, J. Réhault, S. Reiche, M. Ringele, J. Rittmann, L. Rivkin, A. Romann, M. Ruat, C. Ruder, L. Sala, L. Schebacher, T. Schilcher, V. Schlott, T. Schmidt, B. Schmitt, X. Shi, M. Stadler, L. Stingelin, W. Sturzenegger, J. Szlachetko, D. Thattil, D. M. Treyer, A. Trisorio, W. Tron, S. Vetter, C. Vicario, D. Voulot, M. Wang, T. Zamofing, C. Zellweger, R. Zennaro, E. Zimoch, R. Abela, L. Patthey, H.-H. Braun, SwissFEL: The Swiss x-ray free electron laser. *Appl. Sci.* **7**, 720 (2017).

32. B. I. Erkmen, J. H. Shapiro, Ghost imaging: From quantum to classical to computational. *Adv. Opt. Photonics* **2**, 405–450 (2010).

33. M. P. Edger, G. M. Gibson, M. J. Padgett, Principles and prospects for single-pixel imaging. *Nat. Phys.* **13**, 13–20 (2019).

34. H. Yu, R. Lu, S. Han, H. Xie, G. Du, T. Xiao, D. Zhu, Fourier-transform ghost imaging with hard x rays. *Phys. Rev. Lett.* **117**, 113901 (2016).

35. A. Schori, S. Shwartz, X-ray ghost imaging with a laboratory source. *Opt. Express* **25**, 14822–14828 (2017).

36. A.-X. Zhang, Y.-H. He, L.-A. Wu, L.-M. Chen, B.-B. Wang, Tabletop x-ray ghost imaging with ultra-low radiation. *Optica* **5**, 374–377 (2018).

37. A. M. Kingston, D. Pelliccia, A. Rack, M. P. Olbinado, Y. Cheng, G. R. Myers, D. M. Paganin, Ghost tomography. *Optica* **5**, 1516–1520 (2018).

38. D. Pelliccia, M. P. Olbinado, A. Rack, A. M. Kingston, G. R. Myersd, D. M. Paganin, Towards a practical implementation of x-ray ghost imaging with synchrotron light. *IUCrJ* **5**, 428–438 (2018).

39. O. Sefi, Y. Klein, E. Strizhevsky, I. P. Dolbnya, S. Shwartz, X-ray imaging of fast dynamics with single-pixel detector. *Opt. Express* **28**, 24568–24576 (2020).

40. M. P. Olbinado, D. M. Paganin, Y. Cheng, A. Rack, X-ray phase-contrast ghost imaging using a single-pixel camera. *Optica* **8**, 1538–1544 (2021).

41. K. Shibuya, K. Nakae, Y. Mizutani, T. Iwata, Comparison of reconstructed images between ghost imaging and Hadamard transform imaging. *Opt. Rev.* **22**, 897–902 (2015).

42. Z. Zhang, X. Wang, G. Zheng, J. Zhong, Hadamard single-pixel imaging versus Fourier single pixel imaging. *Opt. Express* **25**, 19619 (2017).

43. M. Yabashi, T. Mochizuki, H. Yamazaki, S. Goto, H. Ohashi, K. Takeshita, T. Ohata, T. Matsushita, K. Tamasaku, Y. Tanaka, T. Ishikawa, Design of a beamline for the SPring-8 long undulator source 1. *Nucl. Instrum. Methods Phys. Res. A* **467–468**, 678–681 (2001).

44. T. Kameshima, A. Takeuchi, K. Uesugi, T. Kudo, Y. Kohmura, K. Tamasaku, K. Muramatsu, T. Yanagitani, M. Yabashi, T. Hatsui, Development of an x-ray imaging detector to resolve 200 nm line-and-space patterns by using transparent ceramics layers bonded by solid-state diffusion. *Opt. Lett.* **44**, 1403–1406 (2019).

45. H. Osawa, T. Kudo, S. Kimura, Development of high-repetition-rate x-ray chopper system for time-resolved measurements with synchrotron radiation. *Jpn. J. Appl. Phys.* **56**, 048001 (2017).

46. Y. Tanaka, T. Hara, H. Kitamura, T. Ishikawa, Timing control of an intense picosecond pulse laser to the SPring-8 synchrotron radiation pulses. *Rev. Sci. Instrum.* **71**, 1268–1274 (2000).

47. C. R. Wie, T. A. Tombrello, T. Vreeland, Dynamical x-ray diffraction from nonuniform crystalline films: Application to x-ray rocking curve analysis. *J. Appl. Phys.* **59**, 3743–3746 (1986).

48. O. Mandelung, M. Schulz, H. Weiss, Eds., *Numerical Data and Functional Relationships in Science and Technology, New Series Group III, Vol. 17, Subvolume a, Intrinsic properties of group VI and III-V, II-VI and I-VII Compounds* (Springer, 1982).

49. D. E. Aspnes, A. A. Studna, Dielectric functions and optical parameters of Si, Ge, GaP, GaAs, GaSb, InP, InAs, and InSb from 1.5 to 6.0 eV. *Phys. Rev. B* **27**, 985–1009 (1983).

50. A. J. C. Wilson, Ed., *International Tables for Crystallography Volume C: Mathematical, Physical and Chemical Tables* (International Union of Crystallography, 1992).
